# Antimicrobial Polyamide-Alginate Casing Incorporated with Nisin and ε-Polylysine Nanoparticles Combined with Plant Extract for Inactivation of Selected Bacteria in Nitrite-Free Frankfurter-Type Sausage

**DOI:** 10.3390/foods10051003

**Published:** 2021-05-04

**Authors:** Kazem Alirezalu, Milad Yaghoubi, Leila Poorsharif, Shadi Aminnia, Halil Ibrahim Kahve, Mirian Pateiro, José M. Lorenzo, Paulo E. S. Munekata

**Affiliations:** 1 Department of Food Science and Technology, Ahar Faculty of Agriculture and Natural Resources, University of Tabriz, Tabriz 51666, Iran; 2 Department of Food Science and Technology, Faculty of Agriculture, University of Tabriz, Tabriz 5166616471, Iran; m.yaghoubi97@ms.tabrizu.ac.ir (M.Y.); poorsharif.l@gmail.com (L.P.); 3 Department of Fisheries, Urmia University, Urmia 5756151818, Iran; sh.aminnia@urmia.ac.ir; 4 Department of Food Engineering, Faculty of Engineering, Aksaray University, Aksaray 68100, Turkey; hibrahimkahve@gmail.com; 5 Centro Tecnológico de la Carne de Galicia, Parque Tecnológico de Galicia, Avda. Galicia n 4, San Cibrao das Viñas, 32900 Ourense, Spain; mirianpateiro@ceteca.net (M.P.); jmlorenzo@ceteca.net (J.M.L.); 6 Área de Tecnología de los Alimentos, Facultad de Ciencias de Ourense, Universidad de Vigo, 32004 Ourense, Spain

**Keywords:** polyamide-alginate film, nanoparticle, nisin, ε-polylysine, frankfurter, plant extract, active packaging

## Abstract

The effects of combining a polyamide-alginate casing incorporated with nisin (100 ppm and 200 ppm) and ε-polylysine (500 ppm and 1000 ppm) nanoparticles and a mixed plant extract as ingredient in sausage formulation (500 ppm; composed of olive leaves (OLE), green tea (GTE) and stinging nettle extracts (SNE) in equal rates) were studied to improve the shelf life and safety of frankfurter-type sausage. The film characteristics and microbiological properties of sausage samples were evaluated. Sausage samples were packaged in polyethylene bags (vacuum condition) and analysed during 45 days of storage at 4 °C. Control sausages were also treated with 120 ppm sodium nitrite. Polyamide-alginate films containing 100 ppm nisin and 500 ε-PL nanoparticles had the highest ultimate tensile strength compared to other films. However, 100 ppm nisin and 500 ε-PL nanoparticles decreased water vapour permeability of films. The results also revealed that nisin nanoparticles had significantly (*p* < 0.05) low inhibitory effects against *Escherichia coli*, *Staphylococcus aureus*, molds and yeasts and total viable counts compared to control and ε-PL nanoparticles. Furthermore, 1000 ppm ε-PL nanoparticles displayed the highest antimicrobial activity. Based on the obtained results, the films containing ε-PL nanoparticle could be considered as a promising packaging for frankfurter-type sausages.

## 1. Introduction

Frankfurter-type sausages are a kind of emulsified meat products, which because of their high techno-functional components (bioavailable vitamins (B), essential amino acids, fatty acids, zinc, and heme-iron), ready to eat (RTE) product status, flavour acceptance and low cost, are widely consumed [1,2,3]. However, contamination in meat products like sausages, especially by foodborne bacteria, is the main concern of meat producers [4]. Moreover, frankfurter-type sausages with high fat content are sensitive to oxidation which leads to a reduction in quality (flavour and texture) during storage [5,6], hence researchers endeavour to reduce contamination in sausages using natural antimicrobials and antioxidants [7,8,9].

Increasing the safety of RTE products like sausages could be achieved using antimicrobial packaging [10,11,12,13,14]. Using natural antimicrobial compounds in packaging structures displays higher efficacy in comparison to direct use in the food matrix as an ingredient. The higher efficacy of active antimicrobial packaging may be attributed to the exposure of the food surface (where the risk of contamination is high) to antimicrobial compounds [15,16,17].

Polysaccharides like alginate due to their special colloidal properties such as film-forming, thickening, emulsion stabilizing agent, and gel producing are widely used as a biopolymer film or coating compounds [18]. For instance, sodium alginate has received great consideration due to promising properties in combination with calcium ions as delivery systems for active compounds [19,20,21,22,23]. In this regard, the U.S. Food and Drugs Administration (FDA) has indicated that alginate polymer has the Generally Recognized As Safe (GRAS) status for food use [24]. Furthermore, Surendhiran et al. [25] revealed that phlorotannin encapsulated in a alginate/poly(ethylene oxide) composite film inactivated *Salmonella* spp. and increased the shelf life of chicken meat.

Nisin is a nontoxic and stable bacteriocin produced from *Lactococcus lactis* with authorized use in food [26]. Nisin has strong antimicrobial properties against several spoilage bacteria in meat and meat products such as *Clostridia* and *Bacilli* spores [27,28]. In this regard, Churklam et al. [29] evaluated effects of carvacrol in combination with nisin on sliced Bologna sausage and they noticed that nisin and carvacrol inhibited microbial growth compared to control samples. Furthermore, other authors have reported similar results for nisin in ready-to-eat Yao meat products [30], pork loin [31], frankfurter-type sausage [6] and fresh sausage [32].

ε-Polylysine (ε-PL) is one of the most well-known natural components with high antimicrobial properties against a wide spectrum of bacteria like Gram-positive, Gram-negative bacteria (*Clostridium perfringens, Staphylococcus aureus* and *E. coli*), and yeast and molds [33]. ε-PL has high thermo-stability and is widely utilized in meat industry products like chilled beef [34], and frankfurter type sausage [35] as preservative. Furthermore, antimicrobial efficacy of ε-PL could be increased in combination with plant extracts [35]. Natural plant extracts like olive leaves (OLE), green tea (GTE) and stinging nettle (SNE) extracts are a good source of phenolic compounds with strong antimicrobial and antioxidant properties [36,37,38]. In OLE, the major polyphenols are oleuropein, oleuropein, hydroxytyrosol, tyrosol, luteolin-7-*O*-glucoside, apigenin-7-*O*-glucoside, *p*-coumaric acid, ferulic acid [39]. In the case of GTE the main polyphenols are catechin and its derivatives are epicatechin gallate, epigallocatechin gallate, and epigallocatechin [40]. The phenolic composition of SNE is comprised of flavonoids (kaempferol, rutin, isorhamnetin, and quercetin,) and phenolic acids (*p*-coumaric acid and ferulic acid) [41], making these plant materials rich sources of bioactive and nutrients compounds with potential ability as nitrite substitutes in frankfurter-type sausages [36].

The antimicrobial effect of combined ε-polylysine and nisin with natural antioixdants in frankfurter-type sausage has been reported [6]. To the best of our knowledge, there are no previous studies on the effect of active packaging containing ε-polylysine nanoparticles (ε-PLN) and nisin nanoparticles (NN) on the quality properties and stability of frankfurter-type sausage during storage. The aim of present study was thus to evaluate the effect of active polyamide-alginate films containing nisin and ε-PL nanoparticles in combination with plant extracts on quality and shelf life of frankfurter-type sausages.

## 2. Materials and Methods

### 2.1. Materials

All chemical components and microbial media were of analytical grade (purity >99%) and purchased from Merck (Darmstadt, Germany). Food-grade nisin (Nisaplin, 5000 IU/mL, and ε-polylysine powder (5000 IU/mL,) were purchased from Danisco (Copenhagen, Denmark) and FoodChemand (Shanghai, China), respectively. The high molecular mass chitosan powder (molecular weight 3.1 × 105 g mol^−1^; 95% deacetylation degree) was also purchased from Sigma-Aldrich (Saint Louis, MO, USA). The polyamide film was acquired from Besharat Company (Tabriz, Iran).

### 2.2. Preparation of Plant Extract

The olive leaves (OLE), green tea (GTE) and stinging nettle (SNE) extracts were obtained following the procedure described by Ebrahimzadeh et al. [42] with some modifications. Plant leaves firstly dried in an oven (40 °C) for 48 h and sifted through sieves with 14-inch mesh. Then, 50 g of dried powder and 500 mL of ethanol solution (95%) in Erlenmeyer flask were mixed by magnetic stirrer at room temperature for 48 h. The ethanol in mixture was evaporated at 40 °C in a rotary evaporator after filtering the mixture through a Whatman No 1 filter paper. Equal amounts of GTE, SNE and OLE were prepared for further use in frankfurter-sausage samples.

### 2.3. Preparation of ε-Polylysine Nanoparticle (ε-PLN) and Nisin Nanoparticle (NN)

The stock solution of nisin and ε-PL were prepared according to method described by Alirezalu et al. [35] as follows: 2 g from each of these compounds were separately solubilized in 2% glacial acetic acid solution (200 mL) at 60 °C and filtered through 0.45 μm membrane filter (for sterilization) (Minisart NML, Sartorius, New York, NY, USA). The nanoparticles of nisin and ε-PL were produced according to the method described by Das et al. [43], and Bernela et al. [44] with some modifications. Calcium chloride and ε-PL solution (1:20, *v*/*v*) were mixed together. Then, this solution was mixed with 58.75 mL of sodium alginate (0.63 mg/mL), 12.5 mL of chitosan solution, and 6.25 mL of Pluronic F-68 (1 mg/mL). The final mixture was shaken slowly for 3.5 h (at room temperature). The nanoparticles of ε-PL were obtained after centrifugation (15,000× *g*) (at 4 °C for 0.5 h) and freeze-drying process. The similar technique was utilized for preparation of nisin nanoparticles.

### 2.4. Preparation of Active Antimicrobial Film

The polyamide-alginate films were produced according to the procedures described in [24,45]. Mechanical stirring was used to dissolve 3 g of sodium alginate in 200 mL of sterile deionised water for 30 min (70 °C). After that, for improving films characteristics (increase flexibility and decrease brittleness), glycerol (0.44 g/g of alginate) as a plasticizer was added. Mechanical stirring was utilized for dissolving glucono δ-lactone (5.4 g/g calcium carbonate) with calcium carbonate (0.03 g/g alginate) in 50 mL of distilled water. Then, for calcium alginate films production, the solution was dispersed at 150 mL sodium alginate solution. The ε-PLN (500 and 1000 ppm) and NN (100 and 200 ppm) were added to the mixture and homogenized for 3 min (13,500 rpm at 25 °C) and for integrate absorption of water and gelation of alginate, the attained solution was mixed slowly for 12 h by using mechanical stirring. After that, Petri dishes (10 cm in diagonal) were filled with 10 mL of the solution and dried in an oven (12 h at 45 °C). The Petri dishes were held in desiccators before peeling the films. Finally, polyamide and calcium alginate films were attached together to polyamide-alginate films.

### 2.5. Mechanical Properties

Mechanical properties of calcium alginate films including tensile strength (TS) and elongation at break (%E) were analysed following the ASTM procedure D882-91 [46]. The films were cut into 6 × 0.5 cm and were conditioned by saturated solution of calcium nitrite (RH = 55%) inside a desiccator for 24 h at room temperature. Initial grip separation with 50 mm and cross-head speed with 2 mm/min was used at this study with five replicates for mechanical analysis from each film samples.

### 2.6. Water Vapor Permeability (WVP)

The ASTM procedure E96-95 was utilized for gravimetrically evaluation of calcium alginate film WVP [46]. The calcium alginate films were sealed onto cups (2 cm diagonal and 10 cm height) with 3 g calcium sulfate and before hold inside desiccator containing saturated solution of potassium sulfate (RH = 97%) the cups were weighted. Finally, to attain a 97% RH gradient on the films the desiccator was placed inside the oven (25 °C). The cups were weighted twice a day (12 h to 12 h) for six days and the results were analysed by Fick and Henry’s laws as follows:

(1) $$WVP\left(g\;\mathrm{mm}/m2\;\mathrm{day}\;\mathrm{kPa}\right)=\frac{\mathrm{The}\;\mathrm{rate}\;\mathrm{of}\;\mathrm{water}\;\mathrm{vapor}\;\mathrm{transmission}\;\times \;\mathrm{film}\;\mathrm{thickness}\;\left(\mathrm{mm}\right)}{\mathrm{differential}\;\mathrm{vapor}\;\mathrm{pressure}\;\mathrm{of}\;\mathrm{water}\;\mathrm{through}\;\mathrm{the}\;\mathrm{film}}$$

### 2.7. Preparation of Frankfurters

Each repetition of frankfurter processing was carried out with beef from different animals. The same ingredients and formulation were used in the three batches during three successive days (3 treatments × 4 time periods × 3 repetitions × 3 runs). A local meat processing factory was utilized for sausage production. Frankfurter-type sausage formulation (g/kg) was comprised of 0.4 sodium ascorbate, 15 salt, 81.5 starch and other dry materials, 120 soybean oil, 3.5 polyphosphate sodium, 20 seasoning, 210 ice/water, 0.5 mixed plant extract, and 550 of beef meat. Beef meat was cut into cubes with 3 mm size and homogenized with half of the ice/mixed extract (500 ppm), salt (NaCl) and sodium polyphosphate in a cutter (EX3000 RS, Kilia, Schönkirchen, Germany) at 10 °C for 12 min. After that, other ingredients including seasoning, starch, and sodium ascorbate were added slowly into the mix and homogenized for 1 min. Finally, half of ice/mixed extract (final mixed extract concentration of 500 ppm) and microbial suspensions (10^3^ CFU/g), along with remaining components were added and mixed for about 120 s. The sausages were stuffed mechanically (VF50, Handtmann, Biberach, Germany) into antimicrobial polyamide-alginate films before steam cooking (1.5 h at 80–85 °C).

**Figure 1:**
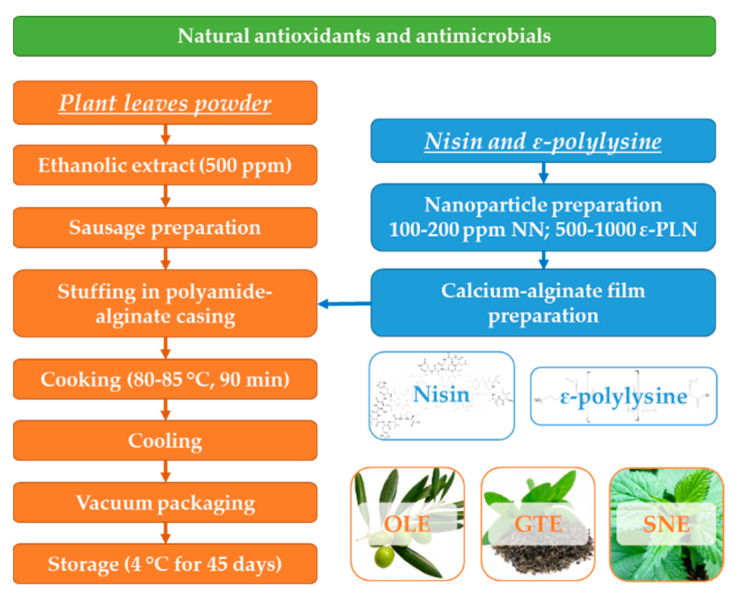
Schematic diagram for frankfurter-type sausage production.

Sausages were quickly chilled with a cold-water shower, packaged in vacuumed condition in polyethylene bags and stored at 4 °C. The following five treatments were prepared: control with polyamide-alginate films without nanomaterials; samples stuffed in the polyamide-alginate films incorporated with 100 and 200 ppm NN, and samples stuffed in the polyamide-alginate films incorporated with 500 and 1000 ppm ε-PLN. Film characteristics and microbial counts were analysed at 0, 15, 30, and 45 days of refrigerated (4 ± 1 °C) storage. A schematic illustration to show the overall workflow is presented in Figure 1.

### 2.8. Microbiological Properties

Microbiological properties of sausage packaged in polyamide alginate films were analysed as follows: 225 mL of 0.1% (*w*/*v*) peptone water and 25 g of samples were homogenized using sterile lab-blender (Paddle Lab Blender, Neutec, Farmingdale, NY, USA) for 3 min. 0.1% of sterile peptone water was also used for serial dilution production. Brilliant Green agar (BGA, Merck, Darmstadt, Germany), Plate Count agar (PCA, Merck) and Sulfite Polymyxin Sulfadizine (SPS) agar (Merck) were used for enumeration of *E. coli*, total viable count, and *Clostridium perfringens*, respectively, by pour-plate technique. Incubation time and temperature for *E. coli*, total viable count, and *Clostridium perfringens* were 24–47 h at 37 °C, 48–72 h at 30 °C and 24 h at 37 °C, respectively. *Staphylococcus aureus* and yeast and molds were enumerated on Baird Parker agar (BPA, Merck) and Dichloran Rose-Bengal Chloramphenicol (DRBC) agar (Merck) following incubation for 48 h at 30 °C and 5 days at 25 °C, respectively. The microbiological results were reported as Log10 CFU/g of sausage samples.

### 2.9. Statistical Analysis

The experimental data resulted from 3 treatments × 4 time periods × 3 repetitions × 3 runs were analysed using the statistical software SAS (v.9, SAS Institute Inc., Cary, NC, USA). Normal distribution and variance homogeneity had been previously tested (Shapiro-Wilk). Random block design was utilized for evaluation of microbiological data, considering a mixed linear model, including replication as a random effect, and different treatments and storage period as fixed impacts. One-way ANOVA was also utilized for mechanical properties and WVP, and Tukey’s test for means comparison (statistical significance at *p* < 0.05 value) and results were expressed as mean values ± standard error in all figures and tables.

## 3. Results and Discussion

### 3.1. Mechanical Properties

The mechanical properties of calcium alginate films including elongation at break (E%) and tensile strength (TS) are shown at Table 1. The results showed that tensile strength of calcium alginate films ranged between 63–67 MPa. Added NN and ε-PLN affected significantly (*p* < 0.05) the tensile strength of films. Pranoto et al. [47] showed that tensile strength of calcium alginate films could decrease by adding garlic oil which may be caused by its hydrophobic properties. In our experiment, tensile strength in calcium alginate films incorporated with 500 ppm ε-PLN was higher than other films (Table 1). Conversely, ε-PLN (500 ppm ε-PLN and 1000 ppm ε-PLN) could significantly (*p* < 0.05) decrease the tensile strength of the films. This fact could be due to the high ε-PLN content, which can reduce tensile strength of the films. Benavides et al. [48] also reported similar results.

**Figure 2:**
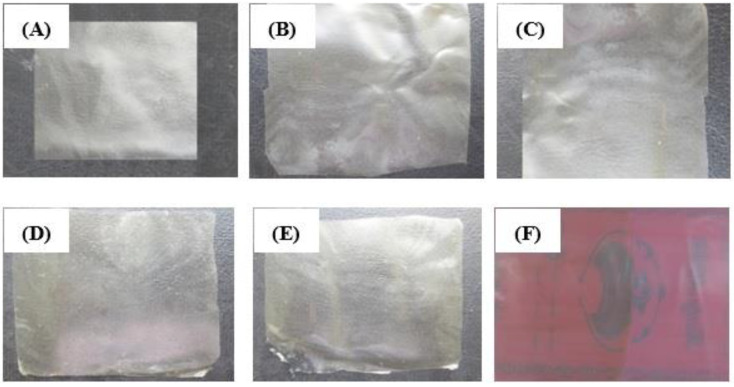
Calcium alginate films and polyamide-alginate film incorporated with NN and ε-PLN. (**A**) Calcium alginate film, (**B**) Calcium alginate film + 100 ppm NN, (**C**) Calcium alginate film + 200 ppm NN, (**D**) Calcium alginate film + 500 ppm ε-PLN, (**E**) Calcium alginate film + 1000 ppm ε-PLN, (**F**) Polyamide-alginate film.

The results also showed that elongation at break (E%) in control films were higher than other films. Incorporating nanoparticles decreased the flexibility of the films, therefore, elongation at break were also decreased in the films. Moreover, link between alginate, calcium and chitosan decreased the flexibility and E% of the films. The results of this study are in agreement with those reported by Guiga et al. [49] in polyamide films. The authors indicated that added nisin in polyethylene and polyamide films could decrease E% of the films from 271% to 130%. The appearance of the active films used are presented in Figure 2.

### 3.2. Water Vapor Permeability (WVP)

**Figure 3:**
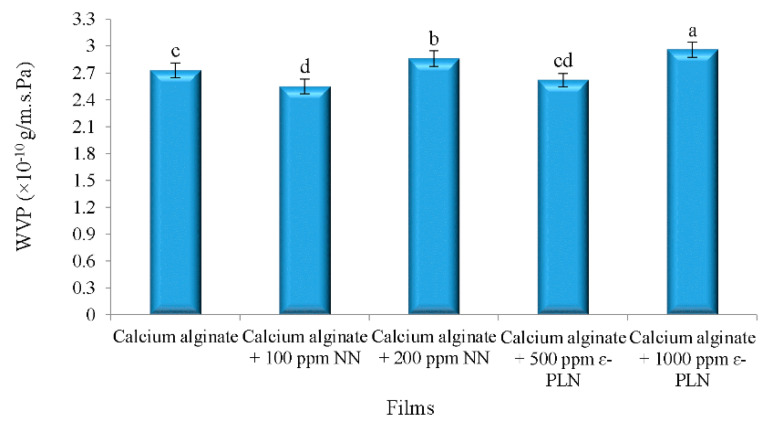
Water vapour permeability of calcium alginate films incorporated with nisin and ε-PL nanoparticles. ^a–d^ Mean values among films not followed by a common letter differ significantly (*p* < 0.05).

The effects of nanoparticles in WVP of calcium alginate films are shown in Figure 3. According to Barzegaran et al. [50], calcium alginate films have lower water vapour permeability (6.16 × 10^–7^ g/m.s.Pa) in comparison to other polymers. Therefore, for production of films with antimicrobial activities with low water vapour permeability, calcium alginate films were produced. The results showed that adding 100 ppm of NN in calcium alginate films decreased WVP of the films, whereas incorporating 200 ppm of NN increased the WVP of the films.

Sodium alginate films can be considered as an edible casing because of its hydrophilic properties (low resistant against moisture) and mechanical stability [51]. Therefore, favouring the gel consistency in films (by adding calcium) can improve water vapour permeability of the films [52].

WVP of the alginate films were decreased by adding 500 ppm ε-PLN. The interaction between amines (chitosan) and carboxyl components (alginate) may be the main reason for the lower WVP in films. Intermolecular gaps and porous microstructure of films matrix significantly affect the permeability of the films. Turhan and Şahbaz [53] showed that plasticizers can increase water vapour permeability of the films by increasing intermolecular gaps. As shown in Figure 3, WVP of films incorporated with 1000 ppm ε-PLN and 200 ppm NN were significantly increased whereas the WVP of films with 500 ppm ε-PLN and 100 ppm NN were decreased. These results may be caused by increasing and decreasing intermolecular gaps, respectively.

### 3.3. Microbiological Properties

Total viable count in all sausage samples (*p* < 0.05) increased during storage. The polyamide-alginate films incorporated with 1000 ppm ε-PLN and 200 ppm NN showed (*p* < 0.05) higher inhibitory effects against TVC. Furthermore, polyamide-alginate films incorporated with ε-PLN presented higher antimicrobial effects compared to polyamide-alginate films with NN. The antimicrobial effects of ε-PL against wide spectrum of microorganisms (Gram-positive, Gram-negative, and fungus) compared to nisin may be the main reason that explains the higher antimicrobial properties of ε-PLN.

**Figure 4:**
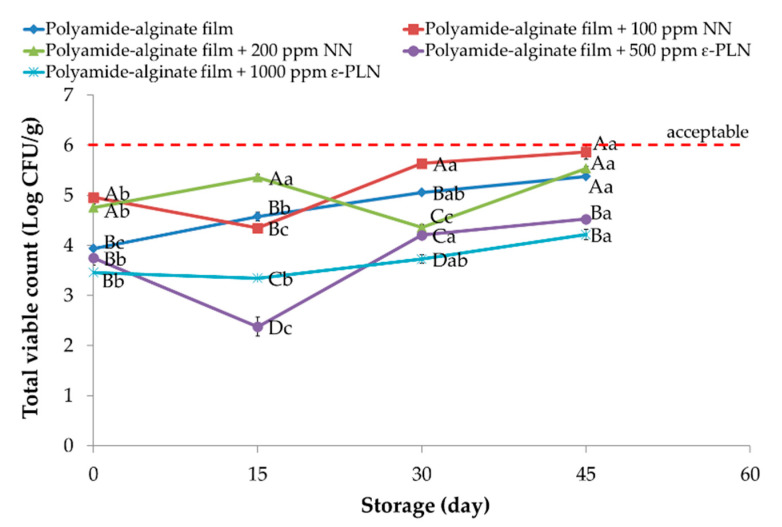
Total viable count of sausage samples packaged in polyamide-alginate films during refrigerated storage. ^A–D^ Mean values among treatments not followed by a common letter differ significantly (*p* < 0.05). ^a–c^ Mean values during storage not followed by a common letter differ significantly (*p* < 0.05).

TVC of frankfurter sausages packaged in polyamide-alginate films with 500 ppm ε-PLN and 100 ppm NN ranged between 5.54 and 5.87 Log CFU/g at the end of storage period (Figure 4). It is worth mentioning that the borderline for microbiological acceptability in meat products (especially due to odour changes) is around 6 Log CFU/g [54,55]. Conversely, TVC in sausage samples packaged in films with 1000 ppm ε-PLN and 200 ppm NN reached 4.22 Log CFU/g and 4.53 Log CFU/g, respectively. Therefore, the results showed that polyamide-alginate films incorporated with 500 and 1000 ppm ε-PLN could (*p* < 0.05) can increase the shelf life of the frankfurter-type sausages (Figure 4). In this regard, Feng et al. [56] indicated that ε-PL with rosemary extract could significantly decrease TVC and improve sensory properties of chicken breast muscle. Additionally, Alirezalu et al. [35] also indicated that ε-PLN displayed significantly higher inhibitory activity against TVC in comparison ε-PL (free form) in frankfurter-type sausage.

Conversely, de Barros et al. [57] evaluated effects of natural casing incorporated with nisin in vacuum packaged sausage for the control of spoilage microorganisms, and reported an inhibitory effects of nisin against TVC. Similar results supporting the antimicrobial activity of nisin were also reported by Neetoo and Mahomoodally [58] on cold smoked salmon (by using cellulose-based films and coatings incorporated with nisin and potassium sorbate), and Ercolini et al. [59] on beef burgers coated in nisin and packaged in LDPE films.

**Figure 5:**
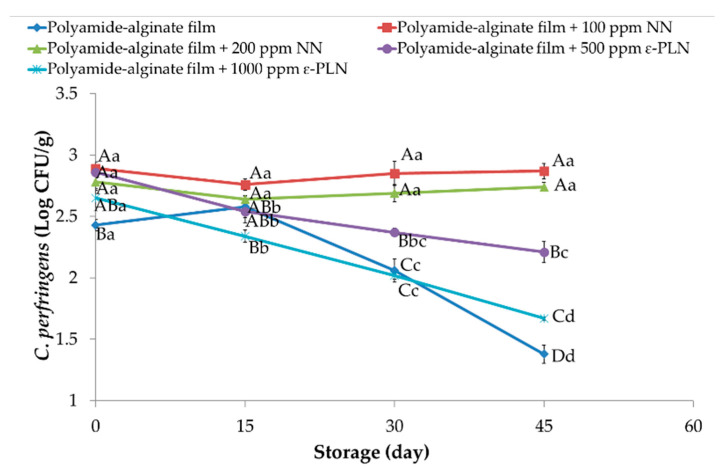
*Clostridium perfringens* of sausage samples packaged in polyamide-alginate films during refrigerated storage. ^A–D^ Mean values among treatments not followed by a common letter differ significantly (*p* < 0.05). ^a–c^ Mean values during storage not followed by a common letter differ significantly (*p* < 0.05).

Regarding the antimicrobial effect of films against *Clostridium perfringens*, counts between 2.43 and 2.86 Log CFU/g in all sausage samples were obtained at the first day of storage (Figure 5). During storage, significant reductions were observed among treatments and at the of storage the treatments control, polyamide-alginate films with 500 and 1000 ppm ε-PLN had lower values than those sausages packaged with polyamide-algine films with 100 and 200 NN. However, significant differences between sausages packaged in polyamide-alginate films containing 1000 ppm ε-PLN and control group after 45 days of refrigerated storage.

Meira et al. [60] evaluated antimicrobial effects of polypropylene/montmorillonite nanocomposites containing different concentration of nisin as antimicrobial active packaging. The authors showed that nisin inhibited the growth of *Clostridium perfringens*, which was stronger in samples with higher concentrations of nisin. In addition, Cé et al. [61] also reported similar results against *Clostridium perfringens* in chitosan films containing nisin. It is important to comment that *Clostridium perfringens* is an anaerobic bacterium that mostly growth in inner sections of the sausage and the antimicrobial were in films that are in contact with the external surface of the samples. Therefore, our results did not show high inhibitory effects against *Clostridium perfringens* in sausage samples.

**Figure 6:**
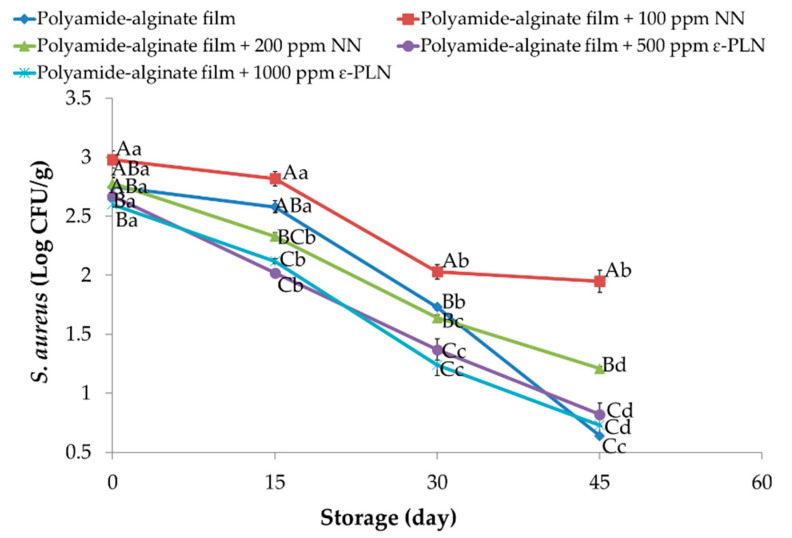
*Staphylococcus aureus* of sausage samples packaged in polyamide-alginate films during refrigerated storage. ^A–C^ Mean values among treatments not followed by a common letter differ significantly (*p* < 0.05). ^a–d^ Mean values during storage not followed by a common letter differ significantly (*p* < 0.05).

*Staphylococcus aureus* in meat and meat products is one of the most important bacteria because of its enterotoxin production [28]. Polyamide-alginate films incorporated with NN and ε-PLN had significant (*p* < 0.05) effects on *Staphylococcus aureus* count (Figure 6). During the refrigerated storage, *Staphylococcus aureus* significantly (*p* < 0.05) decreased in all sausage samples. Our results revealed that *Staphylococcus aureus* counts in samples packaged in polyamide-alginate films incorporated with 500 ppm and 1000 ppm ε-PLN and control sausages were significantly (*p* < 0.05) lower than obtained in other sausages. Elmani [62] evaluated the antimicrobial effects of lysozyme, chitosan, and nisin on Cig kofte (a traditional Turkish raw meatball) and showed that the inhibitory effect of nisin against *Staphylococcus aureus* was higher than chitosan and lysozyme. Our findings agree with data reported by Millette et al. [63] who reported alginate films containing 1000 IU/mL of nisin could decrease 2 Log CFU/cm^2^ of *Staphylococcus aureus* in beef meat after 7 days of storage.

**Figure 7:**
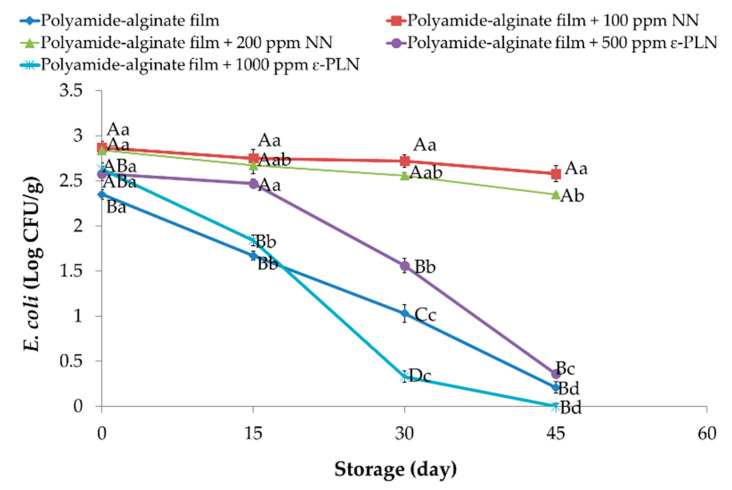
*E. coli* of sausage samples packaged in polyamide-alginate films during refrigerated storage. ^A–D^ Mean values among treatments not followed by a common letter differ significantly (*p* < 0.05). ^a–d^ Mean values during storage not followed by a common letter differ significantly (*p* < 0.05).

ε-PLN significantly (*p* < 0.05) affected *E. coli* in sausage samples (Figure 7). *E. coli* counts continuously decreased in all packaged sausages during storage except for samples packaged with NN (*p* > 0.05) due to lower antimicrobial effects of nisin against Gram-negative bacteria.

The presence of chitosan in nisin nanoparticles’ structure leads to low inhibitory effects of polyamide-alginate films containing nisin against *E. coli*. Our outcomes agree with those reported by Cé et al. [61] who observed that higher concentrations of chitosan had weaker inhibitory effects against *E. coli*. Furthermore, Elmali [62] evaluated the antimicrobial effects of lysozyme, chitosan, and nisin on Cig kofte and reported there were any inhibitory effects against *E. coli* in samples treated with nisin after 72 h of storage. Polyamide-alginate films containing ε-PLN decreased (*p* < 0.05) *E. coli* in sausage samples during the storage time. 

Our results showed that *E. coli* count in sausages packaged in films containing 1000 ppm ε-PLN reached 0 Log CFU/g after 45 days of storage. The high antimicrobial effects of ε-PLN against *E. coli* were also reported by Sun et al. [64] who evaluated the antimicrobial effects of nano-crystalline cellulose films containing ε-PL on fish meat.

**Figure 8:**
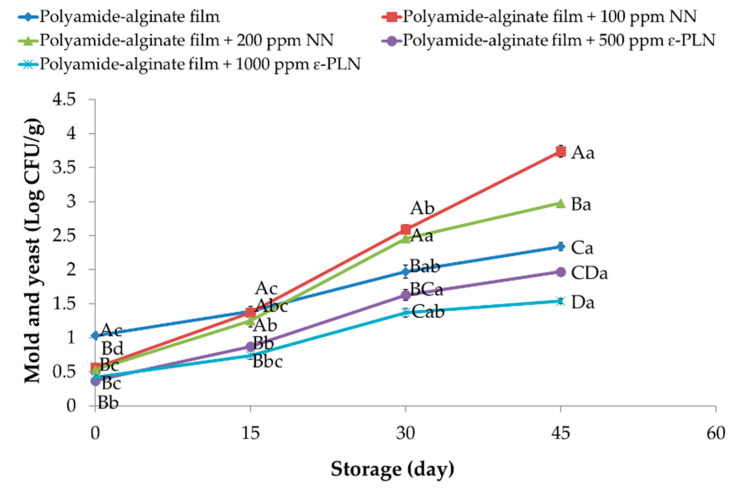
Molds and yeasts of sausage samples packaged in polyamide-alginate films during refrigerated storage. ^A–D^ Mean values among treatments not followed by a common letter differ significantly (*p* < 0.05). ^a–d^ Mean values during storage not followed by a common letter differ significantly (*p* < 0.05).

At day 1, sausage samples packaged in polyamide-alginate films with NN and ε-PLN presented (*p* < 0.05) lower content of molds and yeasts in comparison to control group (Figure 8). During storage, molds and yeasts increased in all sausage samples. At the end of storage time, sausage samples packaged in films containing 100 ppm NN showed the highest counts of molds and yeasts. Our findings agree with data reported by Guerra et al. [65] who evaluated the antimicrobial effects of cellophane containing nisin and reported that bioactive cellophane packaging could be used for controlling microbial growth in chopped meat. Packaged sausage samples in polyamide-alginate containing ε-PLN (500 and 1000 ppm) decreased (*p* < 0.05) the rate on molds and yeasts growth during refrigerated storage. Furthermore, in control samples (with 120 ppm sodium nitrite) molds and yeasts counts until day 30 were within the standard range.

Alirezalu et al. [6] also reported similar results in frankfurter sausages. These authors evaluated the antimicrobial effects of nisin, ε-PL and chitosan and reported a similar inhibitory effect against molds and yeasts in meat products. Moreover, catechins in GTE as a phenolic compound not only can inhibit activity of intracellular enzymes and synthesis of fatty acid and protein but also can damage membrane compounds of molds and yeasts [66].

## 4. Conclusions

Our outcomes showed that polyamide-alginate casing incorporated with ε-PLN and NN with mixed plant extract (same rates of green tea, stinging nettle, and olive leaves extracts) could be potentially used for increasing frankfurter-type sausage shelf life. Sausages with 1000 ppm ε-PLN had significantly higher inhibitory effects against molds and yeasts, *E. coli*, *Staphylococcus aureus*, and total viable counts. Therefore, polyamide-alginate film incorporated with ε-PLN and NN with mixed plant extract could be used usefully for improving frankfurter type sausage quality and shelf life. 

## Figures and Tables

**Table 1:** Mechanical properties of active calcium alginate films.

	Treatments (ppm)					
Properties	Control	100 NN	200 NN	500 ε-PLN	1000 ε-PLN	*p*-Value
σ (MPa)	64.12 ± 2.38 ^b^	66.59 ± 1.37 ^a^	63.15 ± 0.81 ^b^	67.08 ± 2.54 ^a^	63.28 ± 1.78 ^b^	0.03
E (%)	208.34 ± 2.34 ^a^	206.54 ± 1.27 ^ab^	207.65 ± 1.74 ^ab^	205.47 ± 2.65 ^b^	206.35 ± 2.73 ^ab^	0.05

σ: Ultimate tensile strength (CV: 0.54); E: Elongation at break (CV: 1.71). ^a–c^ In each row with different letters differ significantly, (*p* < 0.05).

## Data Availability

The data presented in this study are available on request from the corresponding author.
